# Supplementary material for: Dietary diversity and nutritional status of adults living with HIV during the COVID-19 era
Source: PLoS One. 2024 Jul 19;19(7):e0307427. doi: 10.1371/journal.pone.0307427 (PMC11259258; doi:10.1371/journal.pone.0307427)
Supplement: Attachment — Submitted filename: Peer reviewed plos one.docx [file pone.0307427.s002.docx]

Peer review on the title; Dietary Diversity and Nutritional Status of People Living with HIV (PLHIV) During the COVID-19 Era

Authors: Kasim Abdulai^*1^, Abdul Rauf Alhassan^2^, Safianu Osman Aleboko^1^, Mohammed Doobia Ahmed^3^, Awal Seidu Mohammed^1^, Odei-Asare Fremah Adom^1^, Rhoda Kumah^1^

*^1^Department of Nutrition and Dietetics, University of Cape Coast, Cape Coast, Ghana.*

*^2^Department of Surgery, Tamale Teaching Hospital, Tamale, Ghana*

*^3^Gushegu Midwifery Training College, Gushegu, Ghana*

**Correspondence author:- Kasim Abdulai (email:* [*Kasim.abdulai@ucc.edu.gh*](mailto:Kasim.abdulai@ucc.edu.gh)*)*

**Comments**

1. There are 302 words in your abstract, but more than 300 words are not recommended in this journal.
2. There is no clear objective in the abstract for which this study was desired to be done
3. There is no target age group of study participants in title (child? Adult? Geriatrics?)
4. How many hospitals are there in Central Region of Ghana? How did you select the two hospitals?
5. How many people with HIV/AIDS are there in two hospitals?
6. How did you calculate sample size?
7. Where is/ are your objective/objectives?
8. Are your study participants positive for both HIV/AIDS and COVID-19?
9. Why did you exclude People taking ART for less than 6 months?
10. Was your questionnaire self-administered or interviewer administered?
11. Have you done validity test for your questionnaire that you adopted from FAO? If so the value has to be described.
12. What sampling technique you used to get your study participants? Unclear?
13. In result part, the figures/numbers in table and in body text are not similar
14. In discussion part the possible reason for similarity and difference between your finding and others were not clearly mentioned.
15. The recommendation was not written in its own independent topic rather in continuity with discussion part. It also has to be given based on the significant variables and by whom can it be solved. i.e which variable is to be solved by somebody…
16. You have not described the confidence interval of your outcome variable. So, how can you discuss comparing with different literatures?
17. You have not acknowledged any person or organization which is one component of manuscript
