# Supplementary material for: Dietary diversity and nutritional status of adults living with HIV during the COVID-19 era
Source: PLoS One. 2024 Jul 19;19(7):e0307427. doi: 10.1371/journal.pone.0307427 (PMC11259258; doi:10.1371/journal.pone.0307427)
Supplement: Attachment — Submitted filename: Response to Reviewers.docx [file pone.0307427.s003.docx]

Kasim Abdulai

Department of Nutrition and Dietetics

University of Cape Coast

[Kasim.abdulai@ucc.edu.gh](mailto:Kasim.abdulai@ucc.edu.gh)

The Chief Editor

PLOS ONE

Dear Chief Editor,

**Response to Reviewers**

We would like to express our sincere gratitude for the opportunity to revise our manuscript, titled "Dietary Diversity and Nutritional Status of Adults Living with HIV During the COVID-19 Era,", and for the constructive feedback provided by the academic editor and reviewers. We have carefully considered each comment and have made corresponding revisions to our manuscript. Below, we provide detailed responses to each point raised.

| **Reviewer's Comments** | **Responses** |
| --- | --- |
| 1. There are 302 words in your abstract, but more than 300 words are not recommended in this journal. | DONE |
| 2. There is no clear objective in the abstract for which this study was desired to be done | DONE |
| 3. There is no target age group of study participants in title (child? Adult? Geriatrics?) | INCLUDED |
| 4. How many hospitals are there in Central Region of Ghana? How did you select the two hospitals? | There are six hospitals in the Metropolis that offer ART services. The two hospitals were selected using a computerized random selection method (described on page 5). |
| 5. How many people with HIV/AIDS are there in two hospitals? | The number of active patients at the hospitals were about 1300 for Cape Coast Teaching Hospital and 850 at University of Cape Coast Hospital. We have included it on page 5. |
| 6. How did you calculate sample size? | Sample size determination inserted |
| 7. Where is/are your objective/objectives? | The objective of the study is contained in the abstract and also the last sentence of the introduction. Page 1 for abstract, and page 4 for introduction. |
| 8. Are your study participants positive for both HIV/AIDS and COVID-19? | NO PLEASE. THEY WERE ONLY SERO-POSITIVE FOR HIV AS CONFIRMED |
| 9. Why did you exclude People taking ART for less than 6 months? | Omitting participants who have been on medication for less than six months enables researchers to concentrate on the impacts of prolonged medication use on nutritional and metabolic outcomes. |
| 10. Was your questionnaire self-administered or interviewer administered? | The questionnaire was interviewer administered. |
| 11. Have you done validity test for your questionnaire that you adopted from FAO? | Authors did not find it necessary because the questionnaire has been validated and widely used in the Ghanaian context. This means that its relevance and applicability to the target population have been previously established, and that the tool has demonstrated its effectiveness and appropriateness for research within this specific setting. We refer you to the following citations:   - Matilda, Asante., Benjamin, Frimpong., Freda, Dzifa, Intiful., Portia, Nkumsah-Riverson., Somah, A., Nkansah., Boadiwaa, Ofori-Amanfo., Yaunuick, Y., Dogbe., George, A., Asare. (2021). Food consumption pattern and dietary diversity of a vegetarian population in Ghana. Ghana Medical Journal, doi: 10.4314/GMJ.V55I1.5 - Michael, Akenteng, Wiafe., Charles, Apprey., Reginald, Adjetey, Annan. (2023). Dietary Diversity and Nutritional Status of Adolescents in Rural Ghana. Nutrition and metabolic insights, doi: 10.1177/11786388231158487 - Dickson, A., Amugsi., Zacharie, Tsala, Dimbuene., Pauline, Bakibinga., Elizabeth, W., Kimani-Murage., Tilahun, Nigatu, Haregu., Blessing, Mberu. (2016). Dietary diversity, socioeconomic status and maternal body mass index (BMI): quantile regression analysis of nationally representative data from Ghana, Namibia and Sao Tome and Principe.. BMJ Open, doi: 10.1136/BMJOPEN-2016-012615 |
| 12. What sampling technique you used to get your study participants? | We have included a section on Sampling Procedure, on page 5. |
| 13. In result part, the figures/numbers in table and in body text are not similar | Unfortunately, I could not pick up the portions where there are variations. It would be helpful if you could be specific. |
| 14. In discussion part the possible reason for similarity and difference between your finding and others were not clearly mentioned. | We have included some more explanations. This is found on pages 15 and 16. |
| 15. The recommendation was not written in its own independent topic rather in continuity with discussion part. | We have separated the recommendation from the discussion. This found on page 18. |
| 16. You have not described the confidence interval of your outcome variable. | Authors discussed ORs sometimes expressed in proportions, of significant associations, which were then compared with other findings previously published. |
| 17. You have not acknowledged any person or organization which is one component of manuscript | We have included an acknowledgement section on page 19. |

Sincerely,

**Kasim Abdulai, PhD., RD.**
