# Supplementary material for: Dietary diversity and nutritional status of adults living with HIV during the COVID-19 era
Source: PLoS One. 2024 Jul 19;19(7):e0307427. doi: 10.1371/journal.pone.0307427 (PMC11259258; doi:10.1371/journal.pone.0307427)
Supplement: Attachment — Submitted filename: Response to Reviewers.docx [file pone.0307427.s004.docx]

Kasim Abdulai

Department of Nutrition and Dietetics

University of Cape Coast

[Kasim.abdulai@ucc.edu.gh](mailto:Kasim.abdulai@ucc.edu.gh)

The Chief Editor

PLOS ONE

Dear Chief Editor,

**Response to Reviewers**

We would like to express our sincere gratitude for the opportunity to revise our manuscript, titled "Dietary Diversity and Nutritional Status of Adults Living with HIV During the COVID-19 Era,", and for the constructive feedback provided by the academic editor and reviewers. We have carefully considered each comment and have made corresponding revisions to our manuscript. Below, we provide detailed responses to each point raised.

| **Reviewer's Comments** | **Responses** |
| --- | --- |
| 1. On the line, 117 & 118; you have calculated the sample size to get 217; you rounded to 220. Why? How do you deviate from the formal rule? | Rounding a calculated sample size from 217 to 220 can be considered a deviation from the formal rule of rounding to the nearest whole number. In this case, the decision to round up to 220 instead of rounding to 215 (the nearest whole number below 217) or sticking strictly to 217 depends on several factors and considerations:   1. **Practical Considerations:** Rounding up to 220 may be chosen for practical reasons, such as ensuring that the sample size is easily divisible or manageable in terms of logistical planning for data collection and analysis. 2. **Statistical Power:** Increasing the sample size slightly beyond the calculated value may enhance the statistical power of the study. While the difference between 217 and 220 may seem small, even a modest increase in sample size can improve the study's ability to detect true effects or differences with greater certainty. 3. **Buffer for Attrition or Non-Response:** Rounding up to 220 provides a buffer to account for potential attrition, non-response, or unexpected variability in the sample. This ensures that the study maintains an adequate sample size even if some participants drop out or fail to respond |
| 2. On the line 131, what do you mean to say A total of 220 participants were recruited from two hospitals? | Our study was conducted at two healthcare facilities: Cape Coast Teaching Hospital and University of Cape Coast Hospital, both of which offer ART services. These facilities are situated within Cape Coast Metropolis in the Central Region of Ghana. |
| 3. On the line 133, you have stated the approximated number of active patients were 1300 and 850. What does it mean by active patients? Why did you say approximated? Don’t you know your source and target population? If so, your sample can not represent unknown population. | I apologize for any confusion. When referring to "active patients," it typically means individuals who are actively receiving medical care or treatment within a specific healthcare system or practice. These patients may have ongoing appointments, prescriptions, or other forms of medical interaction within the system.  Regarding the use of "approximately," it is removed**.** |
| 4. On the line 138, you have said to meet the quota… Is your research qualitative or quantitative? | The research is quantitative, and the statement has been modified. Quota is used with respect to sample size distribution among the two facilities based on their respective number of active patients. |
| 5. On line 158, you said information was gathered through the administration of a questionnaire. Was data or information gathered? By whom? | Information was gathered through interviewer administered questionnaire. The statement has been modified. |
| 6. On line 191, what you mean to say multivariable ordinal logistic regression model? Is this model present? | Yes, a multivariable ordinal logistic regression model is a statistical model that is used when analyzing data where the outcome variable is ordinal (i.e., the response variable has ordered categories) and there are multiple predictor variables (i.e., independent variables).  In such a model, the ordinal outcome variable is modeled as a function of one or more predictor variables. The model estimates the relationship between the predictor variables and the odds of an observation falling into a particular category or higher categories of the ordinal outcome variable, while accounting for the ordered nature of the categories.  The ordinal logistic regression model is an extension of binary logistic regression to handle outcomes with more than two ordered categories. It assumes proportional odds, meaning that the effects of the predictor variables on the odds of being in a higher category are assumed to be constant across different levels of the outcome variable. |
| 7. You have not checked model fitness for your study | Model fitness for the study have been checked for both diet quality and nutritional status outcomes. I kindly refer you to lines 275-277, 291-293, and 392-397. |
| 8. You have not checked for confounding factors for which I have not seen the value | In our study, we took several steps to account for potential confounders. Firstly, during the study design phase, we carefully selected variables for inclusion in our analysis based on prior literature and theoretical considerations to minimize the risk of confounding. Furthermore, in our statistical analysis, we employed appropriate methods such as multivariable regression models to adjust for potential confounders that were identified or suspected based on the literature. We ensured that our analysis accounted for their potential influence on the outcome variables. |
| 9. You did not mention the confidence interval of Dietary diversity as well as nutritional status finding that might enable you discuss comparing with that of other findings. | We would like to highlight that our study's outcome variables, dietary diversity and nutritional status, are both assessed based on standardized reference cutoffs widely recognized in the literature. Given that these cutoffs are well-established and widely accepted, there was no need for us to compute confidence intervals for them. Our focus was primarily on examining associations and model fit statistics rather than estimating parameters for these outcome variables. By utilizing standardized reference cutoffs, we ensured consistency and comparability with existing literature, allowing for meaningful discussions and comparisons with other findings. |
|  |  |

Sincerely,

**Kasim Abdulai, PhD., RD.**
