# Supplementary material for: Dietary diversity and nutritional status of adults living with HIV during the COVID-19 era
Source: PLoS One. 2024 Jul 19;19(7):e0307427. doi: 10.1371/journal.pone.0307427 (PMC11259258; doi:10.1371/journal.pone.0307427)
Supplement: Attachment — Submitted filename: Response to reviewers.docx [file pone.0307427.s005.docx]

Kasim Abdulai

Department of Nutrition and Dietetics

University of Cape Coast

[Kasim.abdulai@ucc.edu.gh](mailto:Kasim.abdulai@ucc.edu.gh)

The Chief Editor

PLOS ONE

Dear Chief Editor,

**Response to Reviewers**

We would like to express our sincere gratitude for the opportunity to revise our manuscript, titled "Dietary Diversity and Nutritional Status of Adults Living with HIV During the COVID-19 Era,", and for the constructive feedback provided by the academic editor and reviewers. We have carefully considered each comment and have made corresponding revisions to our manuscript. Below, we provide detailed responses to each point raised.

| Comments | Response |
| --- | --- |
| 1. The abbreviation for COVID-19 should be consistent throughout the document. Avoid using “Covid-19”. Use caps lock as it is an abbreviation and when expanded, give another term/phrase. | Addressed |
| 2. Use the appropriate term “ Background” rather than “Introduction” under the abstraction section. In the next section, use the proper term “Introduction” and remove “background”. | Addressed |
| 3. Throughout your document, you is recommended that you prepare your document alignment in line with PLOS ONE protocol. At the beginning of each paragraph, you have used that central alignment. Please use left alignments. | Addressed |
| 4. Expand all abbreviations used for the first time in the abstract (check if they are used without expanding throughout your document). | Addressed |
| 5. Objectives under the abstract section should be avoided as it currently appears. Merge the aim of your study at the end of the background section under the same section. | Addressed |
| 6. In the results section under the abstract, please clarify that • Aged 40 to 59 years were more likely to exhibit higher dietary diversity (AOR = 1.966, 95% CI: 1.045–4.987). • Factors associated with undernutrition included females (AOR = 1.829, 32, 95% CI: 1.294, 3.872) and first-line ART (AOR = 1.683, 95% CI: 1.282–2.424). | Addressed |
| 7. Add odds ratio for “Employed participants were also more likely to have a high IDDS compared to 31 unemployed participants”. | Addressed |
| 8. Add the conclusion of your study after the results section. Methods In sample size determination, you have used 17% as the proportion of malnutrition. Identify whether it is undernutrition or overnutrition. Explain why you did not consider the nonresponse rate in your study. Discussion Make your writing scientific and neat. Several minor mistakes are noted. Read line by line and you could get them. Mention the study's weakness and strengths Recommendation Merge your recommendation under the conclusions. Please try to adhere to the guidelines. | Regarding the nonresponse rate, we did not explicitly account for it in our sample size calculations due to the typically low attrition rates observed in cross-sectional studies like ours. This assumption was substantiated by our preliminary review of similar studies previously published, which consistently reported minimal issues with participant dropout. Furthermore, in the course of our study, we did not encounter any significant problems related to attrition that would impact the integrity or the statistical power of our findings. Our decision was thus based on both empirical evidence and our specific observational data, ensuring that the sample size was sufficient to achieve the study's objectives without the need for adjusting for nonresponse.  Also, comments on discussion and recommendation have been addressed accordingly. |

Sincerely,

**Kasim Abdulai, PhD., RD.**
