# Supplementary material for: Dietary diversity and nutritional status of adults living with HIV during the COVID-19 era
Source: PLoS One. 2024 Jul 19;19(7):e0307427. doi: 10.1371/journal.pone.0307427 (PMC11259258; doi:10.1371/journal.pone.0307427)
Supplement: Attachment — Submitted filename: Response to reviewers.docx [file pone.0307427.s006.docx]

Kasim Abdulai

Department of Nutrition and Dietetics

University of Cape Coast

[Kasim.abdulai@ucc.edu.gh](mailto:Kasim.abdulai@ucc.edu.gh)

The Chief Editor

PLOS ONE

Dear Chief Editor,

**Response to Reviewers**

We would like to express our sincere gratitude for the opportunity to revise our manuscript, titled "Dietary Diversity and Nutritional Status of Adults Living with HIV During the COVID-19 Era,", and for the constructive feedback provided by the academic editor and reviewers. We have carefully considered each comment and have made corresponding revisions to our manuscript. Below, we provide detailed responses to each point raised.

| Comments | Response |
| --- | --- |
| 1. In the abstract section, expand the acronym COVID-19 properly. Presently, you have expanded it wrongly. | Corrected. |
| 2. Include all subsections of the abstract: Background, methods, results, and conclusions | Done |
| 3. You have not concluded your study findings. Be sure that your conclusion is scientific. | Conclusion has been provided |

Sincerely,

**Kasim Abdulai, PhD., RD.**
